# Risk factors for pneumonia, hepatic encephalopathy, and acute kidney injury in patients with hepatitis B virus–related acute-on-chronic liver failure

**DOI:** 10.3389/fmed.2026.1804550

**Published:** 2026-05-14

**Authors:** Li Wei, Wenjuan Shi, Xuefang Yang, Haiwen Ma, Hong Wan, Xiaoli Zhou

**Affiliations:** Department of Infectious Diseases, Lanzhou Second People's Hospital, Lanzhou, Gansu Province, China

**Keywords:** acute kidney injury, acute-on-chronic liver failure, hepatic encephalopathy, hepatitis B virus, pneumonia

## Abstract

**Background:**

Acute-on-chronic liver failure (ACLF) is a life-threatening syndrome characterized by acute hepatic decompensation in patients with chronic liver disease and is associated with a high short-term mortality rate. Pneumonia, hepatic encephalopathy (HE), and acute kidney injury (AKI) are among the most common and severe complications during hospitalization, yet data regarding their risk factors in hepatitis B virus–related ACLF (HBV-ACLF) remain limited.

**Methods:**

This retrospective observational study consecutively enrolled 132 patients with HBV-ACLF. Demographic characteristics, laboratory parameters, and in-hospital complications were collected. Pneumonia, HE, and AKI were defined according to established guidelines. Univariable and multivariable logistic regression analyses were performed to identify independent risk factors associated with each complication.

**Results:**

A total of 132 patients with HBV-ACLF were included. Pneumonia occurred in 24 patients and was independently associated with older age (OR 1.055, *p* = 0.012) and lower serum albumin levels (OR 0.915, *p* = 0.047). HE developed in 30 patients and was independently associated with elevated serum ammonia (OR 2.403, *p* = 0.047). AKI occurred in 23 patients and was independently associated with older age (OR 1.078, *p* = 0.008) and higher baseline serum creatinine (OR 1.068, *p* = 0.005).

**Conclusion:**

Pneumonia, HE, and AKI in HBV-ACLF are driven by distinct clinical and biochemical risk profiles. Readily available admission variables may facilitate early risk stratification and enable timely, complication-specific interventions, potentially improving clinical outcomes in this high-risk population.

## 1 Introduction

Acute-on-chronic liver failure (ACLF) is a clinical syndrome characterized by acute hepatic decompensation occurring in patients with underlying chronic liver disease (1, 2). It is associated with rapid disease progression, complex pathophysiological mechanisms, and an extremely high short-term mortality rate. In regions with a high prevalence of hepatitis B virus (HBV) infection, particularly in China, HBV-related ACLF represents the most common subtype of liver failure. During hospitalization, patients with ACLF frequently develop severe complications, among which pneumonia, hepatic encephalopathy (HE), and acute kidney injury (AKI) are the most prevalent and are all closely associated with poor short-term outcomes (3–5).

The increased susceptibility to infection in ACLF patients is closely related to multiple mechanisms, including dysregulated systemic inflammatory responses, immune dysfunction, intestinal dysbiosis, and bacterial translocation (5). These alterations collectively impair host defense mechanisms and predispose patients to bacterial infections. In addition, hyperammonemia secondary to liver failure has also been suggested to contribute to an increased risk of infection (6, 7). Previous studies have reported that the incidence of infection in ACLF patients ranges from 39 to 66%, with respiratory tract infections, urinary tract infections, and spontaneous bacterial peritonitis being the most common (8, 9). A retrospective analysis by Cai et al. (8) involving 389 ACLF patients with bacterial infections demonstrated that the most frequent infection sites were the respiratory tract (49.4%), abdominal cavity (37.3%), and urinary system (13.3%). Moreover, a multicenter study indicated that pneumonia is more prevalent among ACLF patients in Asia compared with other regions, with an incidence of approximately 26% (10). Given its insidious onset, rapid progression, and high mortality, identifying the risk factors for pneumonia in ACLF patients is of critical clinical importance for early intervention and outcome improvement.

Hepatic encephalopathy (HE) is another common and severe neurological complication in ACLF, closely related to liver failure, portosystemic shunting, and metabolic disturbances (11, 12). HE is a clinical syndrome characterized by varying degrees of cognitive impairment and central nervous system dysfunction, primarily driven by ammonia metabolism disorders (13, 14). Although the precise mechanisms underlying HE remain incompletely understood, the “ammonia hypothesis” remains the central theory, suggesting that hyperammonemia leads to cerebral energy metabolism impairment and astrocyte swelling (13, 14). Approximately 40% of end-stage liver disease patients develop HE, and multiple studies have demonstrated that HE is strongly associated with increased mortality (15, 16). However, data specifically addressing the risk factors for HE in patients with HBV-related ACLF remain limited.

In addition to liver failure itself, extrahepatic organ failure plays a pivotal role in determining prognosis, with the kidney being one of the most frequently affected organs (17, 18). Studies based on the APASL criteria have reported that 22.8–34% of ACLF patients experience renal dysfunction (17, 19). The development of AKI markedly worsens clinical outcomes in ACLF. AKI in ACLF may result from prerenal, intrinsic renal, or postrenal causes, often occurring in a multifactorial context. Early identification and intervention are crucial for preventing disease progression and improving outcomes in ACLF patients.

Therefore, the present study aimed to systematically investigate the risk factors associated with pneumonia, HE, and AKI in patients with ACLF. By characterizing the clinical features of these complications and their risk factors, we sought to provide evidence for early risk stratification, timely complication recognition, and individualized management strategies in ACLF patients.

## 2 Subjects and methods

### 2.1 Study population

This retrospective observational study consecutively enrolled patients diagnosed with hepatitis B virus–related acute-on-chronic liver failure (HBV-ACLF) who were admitted to Lanzhou Second People’s Hospital between January 2022 and December 2025. All patients were aged ≥18 years and had a confirmed diagnosis of chronic hepatitis B infection with hepatitis B surface antigen (HBsAg) ≥ 6 months. Patients were excluded if they met any of the following criteria: (1) presence of hepatocellular carcinoma or other malignant tumors; (2) prior liver transplantation; (3) human immunodeficiency virus (HIV) infection; (4) pregnancy; or (5) incomplete clinical or laboratory data during hospitalization. A total of 132 patients were enrolled in this study. The mean length of hospital stay in the whole cohort was 31.42 ± 15.68 days. The study was conducted in accordance with the Declaration of Helsinki and was approved by the Institutional Ethics Committee of Lanzhou Second People’s Hospital (SERLL202407). Given the retrospective nature of the study, the requirement for informed consent was waived.

### 2.2 Data collection

Demographic characteristics, clinical manifestations, laboratory parameters, and complications were obtained from electronic medical records. Collected variables included age, sex, routine blood tests, liver function tests, coagulation profiles and renal function indices. Major complications during hospitalization—including pneumonia, HE, and AKI were systematically recorded. All data were independently reviewed by two investigators to ensure accuracy, and discrepancies were resolved by consensus.

### 2.3 Disease definitions

ACLF: the diagnosis of ACLF was established according to the Chinese Guidelines for the Diagnosis and Treatment of Liver Failure (20). ACLF was defined as a clinical syndrome characterized by acute hepatic decompensation in patients with underlying chronic liver disease, manifested by rapidly progressive jaundice and coagulation dysfunction, with serum total bilirubin (TBIL) ≥ 10 times the upper limit of normal (ULN), or an increase ≥17.1 μmol/L per day; and coagulation dysfunction defined as prothrombin activity (PTA) ≤ 40% or international normalized ratio (INR) ≥ 1.5.

Pneumonia: pneumonia was diagnosed based on a combination of radiological, clinical, and laboratory findings (21, 22). The diagnostic criteria required: (1) newly developed or progressive pulmonary infiltrates or consolidation on chest radiography or computed tomography (CT); and (2) at least one of the following clinical or laboratory features: body temperature >38 °C; cough or sputum production; dyspnea; peripheral white blood cell count >10 × 10^9^/L or <4 × 10^9^/L. In our study, all pneumonia cases included were confirmed hospital-acquired.
HE: Hepatic encephalopathy was diagnosed according to the West Haven criteria (23). Diagnosis was based on clinical assessment after exclusion of other causes of altered mental status, such as intracranial infection, cerebrovascular disease, electrolyte imbalance, or drug intoxication.
AKI: AKI was diagnosed according to the International Club of Ascites (ICA) criteria (24). Baseline serum creatinine was defined as the value at hospital admission. AKI was defined as an increase in serum creatinine ≥26.5 μmol/L within 48 h or an increase ≥50% from baseline within 7 days.

All complications (pneumonia, HE, and AKI) were defined as events occurring during hospitalization after the diagnosis of ACLF at admission.

### 2.4 Statistical analysis

Continuous variables were expressed as mean ± standard deviation (SD) or median with interquartile range (IQR), as appropriate, and compared using Student’s *t*-test or the Mann–Whitney *U* test. Categorical variables were presented as counts (percentages) and compared using the chi-square test or Fisher’s exact test. Univariable logistic regression analysis was first performed to identify potential risk factors associated with pneumonia, HE, and AKI. Variables with a *p* < 0.10 in univariable analysis were subsequently entered into multivariable logistic regression models to determine independent risk factors. Results were reported as odds ratios (ORs) with 95% confidence intervals (CIs). All statistical analyses were conducted using SPSS version 26.0 (IBM Corp., NY, United States). A two-sided *p* < 0.05 was considered statistically significant.

## 3 Results

### 3.1 Baseline characteristics of ACLF patients with and without pneumonia

Among the enrolled ACLF patients, 24 developed pneumonia during hospitalization after admission. The mean hospital stay was 31.35 ± 15.87 days in patients without pneumonia and 31.75 ± 15.13 days in those with pneumonia. Patients with pneumonia were significantly older than those without pneumonia (54.21 ± 14.25 vs. 46.34 ± 10.99 years, *p* = 0.017). Sex distribution and the prevalence of cirrhosis were comparable between the two groups. Laboratory analyses showed that serum albumin levels were significantly lower in patients with pneumonia than in those without pneumonia (28.15 ± 5.24 vs. 31.12 ± 7.23 g/L, *p* = 0.024). Although total bilirubin (TBIL) tended to be higher in patients with pneumonia, the difference did not reach statistical significance. Other laboratory parameters, including white blood cell count, platelet count, liver enzymes, renal function indices, coagulation parameters, and serum ammonia, were similar between groups (Table 1).

**Table 1:** Characteristics of ACLF patients with and without pneumonia.

Variable	ACLF patient		*p*
	With pneumonia (*n* = 24)	Without pneumonia (*n* = 108)	
Age, years	54.21 ± 14.25	46.34 ± 10.99	0.017
Sex			0.466
Male	19 (79.2%)	92 (85.2%)	
Female	5 (20.8%)	16 (14.8%)	
Cirrhosis			0.451
Yes	20 (83.3%)	96 (88.9%)	
No	4 (16.7)	12 (11.1%)	
WBC (×10^9^/L)	7.71 ± 3.75	6.73 ± 4.19	0.295
PLT (×10^9^/L)	79.17 ± 52.61	89.90 ± 46.59	0.321
TBIL (μmol/L)	402.58 ± 196.21	357.89 ± 136.95	0.187
ALT (U/L)	333.5 (125.25, 823.5)	407.0 (119.0, 921.25)	0.704
GGT (U/L)	87.5 (53.5, 159.75)	87.5 (59.25, 134.25)	0.998
ALP (U/L)	175.29 ± 97.22	171.49 ± 57.19	0.799
ALB (g/L)	28.15 ± 5.24	31.12 ± 7.23	0.024
Globulin (g/L)	29.59 ± 10.41	30.15 ± 8.21	0.772
BUN (mmol/L)	4.95 (3.93, 5.65)	4.75 (3.70, 6.10)	0.807
NH₃ (μmol/L)	58.73 ± 11.99	68.25 ± 29.19	0.220
AFP (ng/mL)	172.1 (53.3, 342.45)	61.6 (10.92, 281.15)	0.219
INR	1.96 ± 0.56	1.91 ± 0.50	0.705
CRE (μmol/L)	62.5 (51.5, 70.25)	63.5 (54.0, 78.0)	0.390
MELD score	21.64 ± 6.52	21.89 ± 5.27	0.838

WBC, white blood cell count; PLT, platelet count; TBIL, total bilirubin; ALT, alanine aminotransferase; GGT, gamma-glutamyl transferase; ALP, alkaline phosphatase; ALB, albumin; BUN, blood urea nitrogen; NH₃, blood ammonia; AFP, alpha-fetoprotein; INR, international normalized ratio; CRE, serum creatinine, MELD, Model for End-Stage Liver Disease.

### 3.2 Risk factors associated with pneumonia in ACLF patients

Univariate logistic regression analysis demonstrated that older age was significantly associated with the presence of pneumonia in ACLF patients (OR = 1.060, 95% CI: 1.018–1.105, *p* = 0.005), while serum albumin level was inversely associated with pneumonia occurrence (OR = 0.901, 95% CI: 0.820–0.990, *p* = 0.031). Other variables were not significantly associated with pneumonia.

In multivariate logistic regression analysis, age remained an independent risk factor for pneumonia (OR = 1.055, 95% CI: 1.012–1.100, *p* = 0.012), whereas serum albumin continued to show a protective effect (OR = 0.915, 95% CI: 0.830–0.991, *p* = 0.047, Table 2).

**Table 2:** Univariate and multivariate analyses of pneumonia in ACLF patients.

Variables	Univariate analysis			Multivariate analysis		
	OR	95%CI	*p*	OR	95% CI	*p*
Age, years	1.060	1.018–1.105	0.005	1.055	1.012–1.100	0.012
Sex	1.513	0.494–4.633	0.468			
Cirrhosis	0.806	0.442–1.468	0.481			
WBC (×10^9^/L)	1.053	0.955–1.161	0.300			
PLT (×10^9^/L)	0.995	0.985–1.005	0.319			
TBIL (μmol/L)	1.002	0.999–1.005	0.188			
ALT (U/L)	1.000	0.999–1.001	0.646			
GGT (U/L)	1.000	0.997–1.003	0.994			
ALP (U/L)	1.001	0.994–1.007	0.797			
ALB (g/L)	0.901	0.820–0.990	0.031	0.915	0.830–0.991	0.047
Globulin (g/L)	0.992	0.941–1.046	0.770			
BUN (mmol/L)	1.004	0.932–1.081	0.915			
NH3 (umol/L)	0.408	0.113–1.474	0.171			
AFP (ng/mL)	1.023	0.387–2.704	0.964			
INR	1.178	0.508–2.727	0.703			
CRE (μmol/L)	0.998	0.988–1.008	0.688			
MELD score	0.991	0.913–1.077	0.836			

WBC, white blood cell count; PLT, platelet count; TBIL, total bilirubin; ALT, alanine aminotransferase; GGT, gamma-glutamyl transferase; ALP, alkaline phosphatase; ALB, albumin; BUN, blood urea nitrogen; NH₃, blood ammonia; AFP, alpha-fetoprotein; INR, international normalized ratio; CRE, serum creatinine, MELD, Model for End-Stage Liver Disease.

### 3.3 Baseline characteristics of ACLF patients with and without HE

Among the enrolled ACLF patients, 30 patients developed HE (Table 3). The mean hospital stay was 31.87 ± 15.60 days in patients without HE and 29.90 ± 16.11 days in those with HE. Among the 30 patients with HE, 25 were classified as grade I-II and 5 as grade III-IV. There were no significant differences between the HE and non-HE groups in terms of age, sex distribution, or the presence of cirrhosis (all *p* > 0.05). Baseline laboratory parameters, including white blood cell count, platelet count, and liver enzymes were also comparable between the two groups. Notably, Model for End-stage Liver Disease (MELD) scores were significantly higher in patients with HE than in those without HE (23.76 ± 6.58 vs. 21.28 ± 5.03, *p* = 0.030), suggesting a closer association between MELD score and the occurrence of HE in ACLF patients.

**Table 3:** Characteristic of ACLF patients with or without HE.

Variable	ACLF patient		*p*
	With HE (*n* = 30)	Without HE (*n* = 102)	
Age, years	46.57 ± 12.08	48.13 ± 11.99	0.533
Sex			0.661
Male	26 (86.7%)	85 (83.2%)	
Female	4 (13.3%)	17 (16.8%)	
Cirrhosis			0.686
Yes	27 (90.0%)	89 (87.3%)	
No	3 (10.0%)	13 (12.7%)	
WBC (×10^9^/L)	7.12 ± 5.80	6.84 ± 3.50	0.747
PLT (×10^9^/L)	76.23 ± 50.79	91.39 ± 46.46	0.127
TBIL (μmol/L)	400.26 ± 140.74	355.94 ± 151.28	0.154
ALT (U/L)	331.5 (72.0, 854.5)	417.5 (158.75, 968.75)	0.241
GGT (U/L)	78.5 (57.5, 117.75)	91.0 (56.0, 142.25)	0.459
ALP (U/L)	175.13 ± 74.78	171.31 ± 63.33	0.781
ALB (g/L)	29.20 ± 4.89	30.99 ± 7.47	0.219
Globulin (g/L)	32.49 ± 8.63	29.33 ± 8.51	0.077
BUN (mmol/L)	5.0 (4.0, 6.48)	4.7 (3.7, 6.03)	0.151
NH₃ (μmol/L)	70.08 ± 29.47	65.25 ± 26.41	0.457
AFP (ng/mL)	33.63 (7.55, 192.5)	117.0 (14.2, 292.25)	0.180
INR	1.98 ± 0.64	1.90 ± 0.46	0.453
CRE (μmol/L)	64.0 (52.0, 90.0)	63.0 (54.0, 72.0)	0.326
MELD score	23.76 ± 6.58	21.28 ± 5.03	0.030

WBC, white blood cell count; PLT, platelet count; TBIL, total bilirubin; ALT, alanine aminotransferase; GGT, gamma-glutamyl transferase; ALP, alkaline phosphatase; ALB, albumin; BUN, blood urea nitrogen; NH₃, blood ammonia; AFP, alpha-fetoprotein; INR, international normalized ratio; CRE, serum creatinine, MELD, Model for End-Stage Liver Disease.

### 3.4 Univariate and multivariate analysis of risk factors for HE

MELD scores were associated with HE in univariate analysis (OR 1.079, *p* = 0.038), suggesting a potential association with disease severity. However, in the multivariable logistic regression analysis, MELD score was not independently associated with HE after adjustment for confounders (OR 1.031, *p* = 0.597). In multivariate logistic regression analysis, after adjustment for potential confounders, elevated serum ammonia identified as an independent risk factor for HE (OR 2.403, 95% CI 1.009–6.355, *p* = 0.047). Other variables were not independently associated with HE in the multivariate model (see Table 4).

**Table 4:** Univariate and multivariate analyses of HE in ACLF patients.

Variables	Univariate analysis			Multivariate analysis		
	OR	95%CI	*p*	OR	95% CI	*p*
Age, years	0.989	0.956–1.024	0.530			
Sex	0.769	0.238–2.489	0.661			
Cirrhosis	1.315	0.349–4.957	0.686			
WBC (×10^9^/L)	1.016	0.923–1.119	0.745			
PLT (×10^9^/L)	0.993	0.983–1.002	0.129			
TBIL (μmol/L)	1.002	0.999–1.005	0.157			
ALT (U/L)	1.000	0.999–1.000	0.411			
GGT (U/L)	0.999	0.994–1.003	0.528			
ALP (U/L)	1.001	0.995–1.007	0.779			
ALB (g/L)	0.949	0.876–1.028	0.200			
Globulin (g/L)	1.041	0.994–1.090	0.085	1.038	0.987–1.091	0.143
BUN (mmol/L)	1.075	0.990–1.167	0.084	1.091	0.917–1.299	0.327
NH3 (umol/L)	2.374	0.976–5.774	0.057	2.403	1.009–6.355	0.047
AFP (ng/mL)	0.795	0.332–1.901	0.605			
INR	1.340	0.625–2.872	0.452			
CRE (μmol/L)	1.007	0.999–1.015	0.067	0.995	0.975–1.016	0.647
MELD score	1.079	1.004–1.160	0.038	1.031	0.920–1.156	0.597

WBC, white blood cell count; PLT, platelet count; TBIL, total bilirubin; ALT, alanine aminotransferase; GGT, gamma-glutamyl transferase; ALP, alkaline phosphatase; ALB, albumin; BUN, blood urea nitrogen; NH₃, blood ammonia; AFP, alpha-fetoprotein; INR, international normalized ratio; CRE, serum creatinine, MELD, Model for End-Stage Liver Disease.

### 3.5 Baseline characteristics of ACLF patients with and without AKI

Among the 132 patients with HBV-related ACLF, 23 (17.4%) developed AKI during hospitalization. The mean hospital stay was 32.75 ± 15.59 days in patients without AKI and 25.13 ± 14.87 days in those with AKI. As shown in Table 5, patients with AKI were significantly older than those without AKI (55.35 ± 15.09 vs. 46.17 ± 10.63 years, *p* = 0.001). Compared with non-AKI patients, those with AKI had significantly higher white blood cell counts (8.80 ± 6.41 vs. 6.51 ± 3.35 × 10^9^/L, *p* = 0.015), blood urea nitrogen levels (*p* = 0.001), and serum creatinine (*p* < 0.001). No significant differences were observed between the two groups with respect to platelet count, liver enzymes or alpha-fetoprotein levels (all *p* > 0.05).

**Table 5:** Characteristic of ACLF patients with or without AKI.

Variable	ACLF patient		*p*
	With AKI (*n* = 23)	Without AKI (*n* = 109)	
Age, years	55.35 ± 15.09	46.17 ± 10.63	0.001
Sex			0.142
Male	17 (73.9%)	94 (86.2%)	
Female	6 (26.1%)	15 (13.8%)	
Cirrhosis			0.580
Yes	21 (91.3%)	95 (87.2%)	
No	2 (8.7%)	14 (12.8%)	
WBC (×10^9^/L)	8.80 ± 6.41	6.51 ± 3.35	0.015
PLT (×10^9^/L)	89.91 ± 50.11	87.53 ± 47.42	0.829
TBIL (μmol/L)	389.77 ± 188.06	361.00 ± 140.69	0.404
ALT (U/L)	356.0 (85.0, 984.0)	407.0 (126.5, 904.0)	0.653
GGT (U/L)	87.0 (56.0, 180.0)	88.0 (59.0, 130.5)	0.511
ALP (U/L)	163.26 ± 64.54	174.06 ± 66.23	0.477
ALB (g/L)	29.36 ± 5.90	30.84 ± 7.19	0.359
Globulin (g/L)	30.76 ± 11.70	29.90 ± 7.87	0.667
BUN (mmol/L)	6.4 (3.9, 14.7)	4.5 (3.7, 5.75)	0.001
NH₃ (μmol/L)	88.54 ± 49.48	62.83 ± 19.38	0.088
AFP (ng/mL)	47.2 (9.87, 261.2)	115.0 (11.3, 286.9)	0.507
INR	1.87 ± 0.55	1.93 ± 0.50	0.606
CRE (μmol/L)	76.0 (68.0, 145.0)	61.0 (52.0, 70.0)	<0.001
MELD score	25.64 ± 8.56	21.05 ± 4.24	0.019

WBC, white blood cell count; PLT, platelet count; TBIL, total bilirubin; ALT, alanine aminotransferase; GGT, gamma-glutamyl transferase; ALP, alkaline phosphatase; ALB, albumin; BUN, blood urea nitrogen; NH₃, blood ammonia; AFP, alpha-fetoprotein; INR, international normalized ratio; CRE, serum creatinine, MELD, Model for End-Stage Liver Disease.

### 3.6 Univariate and multivariate analyses of factors associated with AKI

Univariate logistic regression analysis identified older age (OR = 1.073, 95% CI: 1.027–1.120, *p* = 0.002), higher white blood cell count (OR = 1.123, 95% CI: 1.013–1.245, *p* = 0.028), elevated blood urea nitrogen (OR = 1.461, 95% CI: 1.160–1.842, *p* = 0.001), and increased serum creatinine (OR = 1.053, 95% CI: 1.024–1.084, *p* < 0.001) as factors significantly associated with the occurrence of AKI in ACLF patients.

However, in the multivariable model, only age and serum creatinine remained independently associated with AKI, while white blood cell count and blood urea nitrogen were no longer significant after adjustment. In the multivariate logistic model, after adjustment for potential confounders, age (OR = 1.078, 95% CI: 1.020–1.139, *p* = 0.008) and serum creatinine (OR = 1.068, 95% CI: 1.020–1.118, *p* = 0.005) remained independent risk factors for AKI in patients with ACLF (Table 6). Other variables, including liver function markers and serum ammonia, were not independently associated with AKI (*p* > 0.05).

**Table 6:** Univariate and multivariate analyses of AKI in ACLF patients.

Variables	Univariate analysis			Multivariate analysis		
	OR	95%CI	*p*	OR	95% CI	*p*
Age, years	1.073	1.027–1.120	0.002	1.078	1.020–1.139	0.008
Sex	2.212	0.752–6.503	0.149			
Cirrhosis	1.547	0.327–7.328	0.582			
WBC (×10^9^/L)	1.123	1.013–1.245	0.028	1.108	0.928–1.323	0.256
PLT (×10^9^/L)	1.001	0.992–1.010	0.827			
TBIL (μmol/L)	1.001	0.998–1.004	0.402			
ALT (U/L)	1.000	0.999–1.001	0.660			
GGT (U/L)	1.000	0.998–1.003	0.794			
ALP (U/L)	0.997	0.990–1.005	0.474			
ALB (g/L)	0.959	0.880–1.045	0.341			
Globulin (g/L)	1.011	0.961–1.064	0.665			
BUN (mmol/L)	1.461	1.160–1.842	0.001	1.093	0.820–1.456	0.543
NH3 (umol/L)	1.994	0.753–5.280	0.165			
AFP (ng/mL)	0.950	0.357–2.529	0.918			
INR	0.774	0.295–2.031	0.603			
CRE (μmol/L)	1.053	1.024–1.084	<0.001	1.068	1.020–1.118	0.005
MELD score	1.150	1.054–1.254	0.002	0.854	0.713–1.024	0.088

WBC, white blood cell count; PLT, platelet count; TBIL, total bilirubin; ALT, alanine aminotransferase; GGT, gamma-glutamyl transferase; ALP, alkaline phosphatase; ALB, albumin; BUN, blood urea nitrogen; NH₃, blood ammonia; AFP, alpha-fetoprotein; INR, international normalized ratio; CRE, serum creatinine, MELD, Model for End-Stage Liver Disease.

## 4 Discussion

In this study, we systematically investigated the risk factors associated with three major in-hospital complications (pneumonia, HE, and AKI) in patients with HBV-ACLF. Our results demonstrate that these complications are highly prevalent and exhibit distinct yet partially overlapping clinical and biochemical determinants. Specifically, older age and hypoalbuminemia were independently associated with pneumonia, elevated serum ammonia was independently associated with HE, and advanced age and high creatinine at admission were independently associated with AKI. These findings highlight the complex, multi-organ nature of ACLF and underscore the importance of early risk stratification and targeted intervention. From a clinical perspective, identifying readily available admission variables that predict major complications may facilitate earlier preventive strategies, individualized monitoring, and optimized resource allocation in this critically ill population.

Pulmonary infection is one of the most frequent and life-threatening complications in ACLF, often precipitating systemic inflammation, respiratory failure, and further organ dysfunction. Previous studies have reported infection rates relatively high in ACLF patients, with pneumonia being the most common infection site, particularly in Asian cohorts (10). In line with these observations, our study confirmed that pneumonia is a frequent complication in HBV-ACLF and is associated with distinct baseline characteristics. We found that older age and lower serum albumin levels were independently associated with the development of pneumonia. These findings suggest that factors beyond disease severity may contribute to pneumonia risk. However, further studies are needed to validate this observation. Aging is well recognized to be associated with immunosenescence and impaired pulmonary defense mechanisms, while hypoalbuminemia reflects poor nutritional status, impaired hepatic synthetic function, and compromised immune competence (25–29). Compared with prior studies that emphasized inflammatory markers or disease severity scores, our findings highlight the prognostic relevance of host-related vulnerability factors. Clinically, these results suggest that elderly HBV-ACLF patients and those with hypoalbuminemia should be considered a high-risk group for pneumonia, warranting intensified respiratory monitoring, early antimicrobial stewardship, and nutritional support.

HE is a hallmark complication of ACLF and is strongly associated with increased mortality and poor neurological outcomes. Although hyperammonemia has long been considered central to HE pathogenesis, accumulating evidence indicates that HE results from a multifactorial interplay involving liver dysfunction, systemic inflammation, and extrahepatic organ failure (30, 31). In our study, HE was independently associated with elevated serum ammonia levels. These findings are consistent with the classical ammonia hypothesis. Previous studies have variably identified ammonia, infection, renal dysfunction, and inflammatory mediators as risk factors for HE in ACLF (12, 32). Our study demonstrated that serum ammonia is independently associated with HE after adjustment for potential confounders included in the multivariable model. Clinically, these findings suggest that early ammonia-lowering strategies may be beneficial Due to the limited number of patients with severe HE, further analysis of factors associated with HE severity was not performed. Future studies with larger sample sizes are needed to address this issue.

AKI represents one of the most devastating complications of ACLF and markedly worsens short-term survival. In our cohort, AKI was strongly associated with older age and elevated serum creatinine at admission, both of which emerged as independent predictors. This finding is consistent with current guideline recommendations emphasizing early renal risk assessment at admission (33). Advanced age likely reflects reduced renal reserve and increased susceptibility to hemodynamic instability, while elevated creatinine indicates pre-existing or early renal impairment. Previous studies have highlighted systemic inflammation, circulatory dysfunction, and infection as contributors to AKI in liver failure (3, 34). While our findings align with the established importance of renal dysfunction, they further emphasize the role of baseline renal vulnerability rather than liver injury markers alone. However, serum creatinine at admission was independent associated with AKI, although it is also part of the diagnostic criteria, which may introduce potential incorporation bias. Therefore, it should not be interpreted as a purely independent predictive factor. Instead, elevated baseline creatinine is more likely to reflect pre-existing renal impairment or reduced renal reserve, which predisposes patients to AKI during the course of ACLF. The results should be interpreted with caution. From a clinical standpoint, these results support early identification of renal impairment at admission and proactive measures such as hemodynamic optimization, avoidance of nephrotoxic agents, and early nephrology consultation to prevent AKI progression. Notably, some variables that were significant in univariable analyses did not retain significance after adjustment, highlighting the importance of multivariable modeling to account for potential confounding factors.

One important limitation of this study is the absence of formal ACLF grading. To partially account for disease severity, we incorporated the MELD score into our analyses as an objective and widely used surrogate marker of liver dysfunction. However, it should be noted that MELD primarily reflects hepatic function and does not fully capture extrahepatic organ failures, which are essential components of ACLF grading. Therefore, this limitation should be considered when interpreting the results. Several other limitations should be also acknowledged. First, this was a single-center retrospective study, which may limit generalizability and introduce selection bias. Second, dynamic changes in laboratory parameters and complication evolution during hospitalization were not fully captured, potentially underestimating time-dependent associations. Third, the relatively small number of patients in certain subgroups. Future prospective, multicenter studies incorporating ACLF grading systems with longitudinal data collection are warranted to validate our findings and explore causal relationships. Incorporation of dynamic biomarkers and standardized infection severity assessments may further enhance risk prediction accuracy.

## 5 Conclusion

In conclusion, our study provides a comprehensive analysis of the risk factors associated with pneumonia, hepatic encephalopathy, and acute kidney injury in patients with HBV-ACLF. We demonstrate that these complications are driven by distinct clinical and biochemical profiles, reflecting differential pathophysiological mechanisms. Importantly, readily available admission variables—such as age, albumin, ammonia and creatinine—may aid in early risk stratification. These findings have important implications for clinical practice, emphasizing the need for individualized monitoring strategies and early, complication-specific interventions to improve outcomes in this high-risk population.

## Data Availability

The original contributions presented in the study are included in the article/supplementary material, further inquiries can be directed to the corresponding author.

## References

[1] Piano S Mahmud N Caraceni P Tonon M Mookerjee RP. Mechanisms and treatment approaches for ACLF. Liver Int. (2025) 45:e15733. doi: 10.1111/liv.15733 37715608 PMC12036731

[2] Trebicka J Hernaez R Shawcross DL Gerbes AL. Recent advances in the prevention and treatment of decompensated cirrhosis and acute-on-chronic liver failure (ACLF) and the role of biomarkers. Gut. (2024) 73:1015–24. doi: 10.1136/gutjnl-2023-330584, 38527788 PMC11103292

[3] Maiwall R Sharma F. AKI in ACLF: navigating the complex therapeutic puzzle. Expert Rev Gastroent. (2025) 19:165–80. doi: 10.1080/17474124.2025.2456121, 39825627

[4] Romero-Gómez M Montagnese S Jalan R. Hepatic encephalopathy in patients with acute decompensation of cirrhosis and acute-on-chronic liver failure. J Hepatol. (2015) 62:437–47. doi: 10.1016/j.jhep.2014.09.005 25218789

[5] Yang L Wu T Li J Li J. Bacterial infections in acute-on-chronic liver failure. Semin Liver Dis. (2018) 38:121–33. doi: 10.1055/s-0038-1657751 29871019

[6] Thanapirom K Treeprasertsuk S Choudhury A Verma N Dhiman RK Al Mahtab M . Ammonia is associated with liver-related complications and predicts mortality in acute-on-chronic liver failure patients. Sci Rep. (2024) 14:5796. doi: 10.1038/s41598-024-56401-x, 38461166 PMC10924893

[7] Tranah TH Ballester M Carbonell-Asins JA Ampuero J Alexandrino GA Caracostea A . Plasma ammonia levels predict hospitalisation with liver-related complications and mortality in clinically stable outpatients with cirrhosis. J Hepatol. (2022) 77:1554–63. doi: 10.1016/j.jhep.2022.07.014, 35872326

[8] Cai J Zhang M Han T Jiang H. Characteristics of infection and its impact on short-term outcome in patients with acute-on-chronic liver failure. Medicine. (2017) 96:e8057. doi: 10.1097/MD.0000000000008057, 28906399 PMC5604668

[9] Wong F Piano S Singh V Bartoletti M Maiwall R Alessandria C . Clinical features and evolution of bacterial infection-related acute-on-chronic liver failure. J Hepatol. (2021) 74:330–9. doi: 10.1016/j.jhep.2020.07.046, 32781201

[10] Piano S Singh V Caraceni P Maiwall R Alessandria C Fernandez J . Epidemiology and effects of bacterial infections in patients with cirrhosis worldwide. Gastroenterology. (2019) 156:1368–1380.e10. doi: 10.1053/j.gastro.2018.12.005, 30552895

[11] Cordoba J Ventura-Cots M Simón-Talero M Amorós À Pavesi M Vilstrup H . Characteristics, risk factors, and mortality of cirrhotic patients hospitalized for hepatic encephalopathy with and without acute-on-chronic liver failure (ACLF). J Hepatol. (2014) 60:275–81. doi: 10.1016/j.jhep.2013.10.004, 24128414

[12] Badal BD Bajaj JS. Hepatic encephalopathy in acute-on-chronic liver failure. Clin Liver Dis. (2023) 27:691–702. doi: 10.1016/j.cld.2023.03.012 37380292

[13] Br VK Sarin SK. Acute-on-chronic liver failure: terminology, mechanisms and management. Clin Mol Hepatol. (2023) 29:670–89. doi: 10.3350/cmh.2022.0103, 36938601 PMC10366797

[14] Lee G. Hepatic encephalopathy in acute-on-chronic liver failure. Hepatol Int. (2015) 9:520–6. doi: 10.1007/s12072-015-9626-0 26016460

[15] Krishnarao A Gordon FD. Prognosis of hepatic encephalopathy. Clin Liver Dis. (2020) 24:219–29. doi: 10.1016/j.cld.2020.01.004 32245529

[16] European Association for the Study of the Liver. EASL Clinical Practice Guidelines on the management of hepatic encephalopathy. J Hepatol. (2022) 77:807–24. doi: 10.1016/j.jhep.2022.06.001 35724930

[17] Sarin SK Choudhury A Sharma MK Maiwall R Al Mahtab M Rahman S . Acute-on-chronic liver failure: consensus recommendations of the Asian Pacific association for the study of the liver (APASL): an update. Hepatol Int. (2019) 13:353–90. doi: 10.1007/s12072-019-09946-3, 31172417 PMC6728300

[18] Nanchal R Subramanian R Karvellas CJ Hollenberg SM Peppard WJ Singbartl K . Guidelines for the management of adult acute and acute-on-chronic liver failure in the ICU: cardiovascular, endocrine, hematologic, pulmonary, and renal considerations. Crit Care Med. (2020) 48:e173–91. doi: 10.1097/CCM.0000000000004192 32058387

[19] Sarin SK Kedarisetty CK Abbas Z Amarapurkar D Bihari C Chan AC . Acute-on-chronic liver failure: consensus recommendations of the Asian Pacific Association for the Study of the liver (APASL) 2014. Hepatol Int. (2014) 8:453–71. doi: 10.1007/s12072-014-9580-2, 26202751

[20] Liver Failure and Artificial Liver Group CSOI, Severe Liver Disease and Artificial Liver Group CSOH. Guidelines for diagnosis and treatment of liver failure (2024 version). Chin J Hepatol. (2025) 33:18–33. doi: 10.3760/cma.j.cn501113-20241206-00614, 39929681 PMC12899238

[21] Xu Z Zhang X Chen J Shi Y Ji S. Bacterial infections in acute-on-chronic liver failure: epidemiology, diagnosis, pathogenesis, and management. J Clin Transl Hepatol. (2024) 12:667–76. doi: 10.14218/JCTH.2024.00137, 38993512 PMC11233977

[22] Wang N Zheng Y Tao S Chen L. Risk factors and prognosis of pulmonary infection in hepatitis B-related acute-on-chronic liver failure: a retrospective cohort study. BMC Pulm Med. (2025) 25:178. doi: 10.1186/s12890-025-03628-7, 40229810 PMC11995551

[23] Häussinger D Dhiman RK Felipo V Gorg B Jalan R Kircheis G . Hepatic encephalopathy. Nat Rev Dis Primers. (2022) 8:43. doi: 10.1038/s41572-022-00366-6 35739133

[24] Angeli P Gines P Wong F Bernardi M Boyer TD Gerbes A . Diagnosis and management of acute kidney injury in patients with cirrhosis: revised consensus recommendations of the International Club of Ascites. Gut. (2015) 64:531–7. doi: 10.1136/gutjnl-2014-308874, 25631669

[25] Chebib N Müller F Prendki V. Pneumonia of the elderly and its link to oral health. Rev Med Suisse. (2018) 14:2007–11. doi: 10.53738/REVMED.2018.14.626.2007 30422420

[26] Chen Q Wang L Yu W Xi H Zhang Q Chen X . Recommendations for the prevention and treatment of the novel coronavirus pneumonia in the elderly in China. Aging Med (Milton). (2020) 3:66–73. doi: 10.1002/agm2.12113 32661507 PMC7300900

[27] Chalmers JD. The modern diagnostic approach to community-acquired pneumonia in adults. Semin Resp Crit Care. (2016) 37:876–85. doi: 10.1055/s-0036-1592125 27960211

[28] Yao W Sun X Tang W Wang W Lv Q Ding W. Risk factors for hospital-acquired pneumonia in hip fracture patients: a systematic review and meta-analysis. BMC Musculoskel Dis. (2024) 25:6. doi: 10.1186/s12891-023-07123-0, 38166762 PMC10759764

[29] Tian Y Zhu Y Zhang K Tian M Qin S Li X. Relationship between preoperative hypoalbuminemia and postoperative pneumonia following geriatric hip fracture surgery: a propensity-score matched and conditional logistic regression analysis. Clin Interv Aging. (2022) 17:495–503. doi: 10.2147/CIA.S352736, 35444412 PMC9013674

[30] Bernardini AP. Hepatic encephalopathy: pathogenesis. Arq Gastroenterol. (1988) 25:57–61. 3060059

[31] Bai Y Li K Li X Chen X Zheng J Wu F . Effects of oxidative stress on hepatic encephalopathy pathogenesis in mice. Nat Commun. (2023) 14:4456. doi: 10.1038/s41467-023-40081-8, 37488119 PMC10366183

[32] Bajaj JS Silvey S Mullan A Choudhury A Wong F George J . Liver failure, hepatic encephalopathy, and infection contribute to mortality risk in a global cirrhosis cohort. Clin Gastroenterol Hepatol. (2025) 24:453–462.e14. doi: 10.1016/j.cgh.2025.06.021, 40618943 PMC12379001

[33] Khwaja A. KDIGO clinical practice guidelines for acute kidney injury. Nephron Clin Pract. (2012) 120:c179–84. doi: 10.1159/000339789 22890468

[34] Saha R Sharma S Mondal A Sati HC Khan MA Mahajan S . Evaluation of acute kidney injury (AKI) biomarkers FABP1, NGAL, cystatin c and IL-18 in an Indian cohort of hospitalized acute-on-chronic liver failure (ACLF) patients. J Clin Exp Hepatol. (2025) 15:102491. doi: 10.1016/j.jceh.2024.102491 39901974 PMC11787010

